# Nitrooleic Acid Protects against Cisplatin Nephropathy: Role of COX-2/mPGES-1/PGE_2_ Cascade

**DOI:** 10.1155/2015/293474

**Published:** 2015-03-11

**Authors:** Haiping Wang, Zhanjun Jia, Jing Sun, Liang Xu, Bing Zhao, Kezhou Yu, Meng Yang, Tianxin Yang, Rong Wang

**Affiliations:** ^1^Department of Nephrology, Provincial Hospital Affiliated to Shandong University, No. 324 Jingwu Road, Jinan, Shandong 250013, China; ^2^Department of Internal Medicine, University of Utah and Salt Lake Veterans Affairs Medical Center, Salt Lake City, UT 84112, USA

## Abstract

Nitrooleic acid (OA-NO_2_) is an endogenous lipid product which has novel signaling properties, particularly the activation of peroxisome proliferator-activated receptors. The current study aimed to evaluate the protective effects of OA-NO_2_ against cisplatin-induced kidney injury in mice. Mice were pretreated with OA-NO_2_ for 48 h before cisplatin administration, and the cisplatin-caused nephrotoxicity was evaluated. After the cisplatin treatment (72 h), the vehicle-treated mice displayed renal dysfunction, as evidenced by the elevated plasma urea and creatinine, which was consistent with the histological damage, such as tubular necrosis, dilation, protein cast, and desquamation of epithelial cells. In contrast, the severity of the renal dysfunction and histological change were reduced in the OA-NO_2_ pretreated mice. The renal COX-2 and mPGES-1 mRNAs and their respective proteins expression, together with the renal PGE_2_ amounts, were induced by the cisplatin treatment, but their initiation was reduced by OA-NO_2_. Moreover, the circulating TNF-*α*, renal TNF-*α*, IL-1*β*, MCP-1, ICAM-1, and VACAM-1 mRNA levels were higher in the cisplatin-treated mice, compared with the controls, but they were attenuated in the OA-NO_2_ pretreatment group. In summary, the pretreatment with OA-NO_2_ remarkably ameliorated the cisplatin-induced kidney injury in mice, possibly via the inhibition of the inflammatory response, associated with the COX-2/mPGES-1/PGE_2_ cascade.

## 1. Introduction

*cis*-Diamminedichloroplatinum (cisplatin) is a highly effective antineoplastic DNA-alkylating agent, and the cisplatin-based combination chemotherapy regimens are currently used as the front-line therapy in the treatment of a number of cancer types: testicular cancer, cancer of the bladder, epithelial ovarian cancer, endometrial cancer, cancer of the head and neck, ovarian germ cell tumors, advanced cervical cancer, mesothelioma, non-small-cell lung cancer [1], and so forth. The therapeutic effects of cisplatin are significantly improved by its dose elevation. However, the high-dose therapy with cisplatin is limited by the emergence of serious adverse effects, particularly nephrotoxicity [2], which is a frequent adverse result with about 25–35% patients suffering a decline in their renal functions after a single dose of cisplatin [3].

The severity of toxicity in the early clinical trials called into question the use of cisplatin as a chemotherapy agent. Hydration protocols were developed that reduced the nephrotoxicity and allowed an increase of the dose to achieve therapeutic levels [4]. However, even with vigilant hydration, approximately one-third of the patients treated with cisplatin have a transient elevation of blood urea nitrogen levels or other evidence of kidney damage in the days following the cisplatin treatment [5].

Multiple studies have examined the mechanisms of cisplatin-induced kidney injury. The signaling mechanisms, responsible for the cisplatin-induced cytotoxicity, appear to be multifactorial, involving inflammation, oxidative stress, and caspase actions [6]. Indeed, growing evidence suggests that the inhibition of inflammatory gene expression is partially capable of attenuating cisplatin-induced renal injury [7]. However, with the exception of hydration with saline, no specific treatments have been performed against cisplatin-induced nephrotoxicity [4]. Therefore, an urgent need exists to develop new, effective treatments against cisplatin-induced kidney injury.

Nitrated free fatty acids (NO_2_-FA), particularly the nitroalkene derivatives of linoleic acid (nitrolinoleic acid; LNO_2_) and nitrooleic acid (OA-NO_2_), are identified as endogenous molecules with several attractive signaling properties [8, 9]. Nitrooleic acid (OA-NO_2_) and related nitroalkenes are present in normal tissues at nM concentrations and can increase during inflammation to almost *μ*M concentrations, indicating fatty acid nitroalkenes could induce a variety of pharmacological effects [10]. An increasing body of findings attests that nitrated free fatty acid exerts potent anti-inflammatory actions* in vitro* and* in vivo*. Diverse signaling properties have been attributed to nitrooleic acid. In animal models, OA-NO_2_ has demonstrated benefits in hypertension [11], vascular neointimal proliferation [12], obesity with the metabolic syndrome [13], and hyperglycemia in diabetes [14]. Considerable evidence demonstrates that OA-NO_2_ exerts potent anti-inflammatory actions. The pretreatment with OA-NO_2_ inhibits the lipopolysaccharide- (LPS-) induced NF-*κ*B activation both* in vivo* and in isolated macrophages [15]. Moreover, OA-NO_2_ attenuated colonic inflammation and improved the clinical symptoms in experimental inflammatory bowel disease, dependent on the PPAR gamma pathway in mice [16]. We previously demonstrated that nitrooleic acid exhibited protective effects against the renal injury in an ischemia and reperfusion mouse model and a LPS-induced endotoxic model via anti-inflammation and antioxidative stress [17, 18]. The present study seeks to examine the potential therapeutic effects of OA-NO_2_ in cisplatin-caused nephropathy.

## 2. Materials and Methods

### 2.1. Materials

Cisplatin (*cis*-diamminedichloroplatinum (II)) was purchased from Sigma-Aldrich (St. Louis, MO, USA). The TO901317 compound was purchased from the Cayman Chemical Company (Ann Arbor, MI, USA) and dissolved in DMSO. The two regioisomers of OA-NO_2_, 9- and 10-nitrooleic acids, are formed* in vivo* in approximately equal proportions by nitration of oleic acid [19]. Both compounds were purchased from Cayman Chemical (Ann Arbor, MI, USA), dissolved in dimethyl sulfoxide (DMSO), and used as a 1 : 1 mixture of the isomers.

### 2.2. Animal Experiments

Male C57BL/6 mice (8–10-week-old) were maintained on a standard rodent chow with free access to water. The animals were divided into three groups: control (control; *n* = 6), cisplatin-induced kidney injury (cisplatin; *n* = 12), and cisplatin-induced kidney injury with OA-NO_2_ treatment (cisplatin + OA-NO_2_; *n* = 13) groups. Cisplatin was dissolved in saline at a concentration of 2 mg/mL and used to treat the mice by a single intraperitoneal (i.p.) injection (20 mg/Kg), in both the cisplatin and cisplatin + OA-NO_2_ groups. The OA-NO_2_ was dissolved in 100% DMSO at 100 mg/mL. The mice were pretreated for 48 h with DMSO (cisplatin + vehicle) or OA-NO_2_ (cisplatin + OA-NO_2_) at a dose of 2 mg/kg/d via a microosmotic pump (DURECT Corporation, Cupertino, CA, USA). The control mice group received an i.p. injection of saline. After mouse euthanasia (72 h after the cisplatin treatment), blood samples were obtained from the inferior vena cava, and both kidneys were excised. All protocols employing mice were conducted in accordance with the principles and guidance of the Ethics Committee of Shandong University.

### 2.3. Renal Function

Plasma urea (BUN) and creatinine (CREA) levels were determined to assess the renal function. After blood collection, the serum levels of these toxicity markers were measured immediately by using a blood chemistry analyzer.

### 2.4. Renal Histology

Mice kidneys were fixed in 4% paraformaldehyde, embedded in paraffin, sectioned at 4 *μ*m, and stained with hematoxylin and eosin (HE) and periodic acid-Schiff (PAS), respectively, by standard methods. The tissue damage was indicated by tubular lysis, dilation, necrosis, and cast formation and glomeruli congestion and atrophy. And the scoring scale scores (none (—); mild damage (+); moderate damage (++); and severe damage (+++)) are semiquantitative scores given by a pathologist unaware of the type of treatment.

### 2.5. Real-Time RT-PCR

Kidneys were harvested and preserved in the RNAlater solution (Sangong Biotech, China) at −20°C until RNA extraction. The total RNA was isolated by the use of TRIzol (Invitrogen, CA, USA), and cDNA was synthesized through Superscript (TaKaRa, Japan). Real-time RT-PCR was carried out employing a QuantiTect SYBR Green kit (Qiagen, Germany) on an ABI Prism 7500 real-time PCR (RT-PCR) instrument, equipped with the appropriate software (Applied Biosystems, CA, USA). The oligonucleotide sequences (Sangong Biotech, Shanghai, China) used for the real-time PCR were as follows: GAPDH, sense 5′-GTCTTCACTACCATGGAGAGG-3′ and antisense 5′-TCATGGATGACCTTGGCCAG-3′; TNF-*α*, sense 5′-TCCCCAAAGGGATGAGAAG-3′ and antisense 5′-CACTTGGTGGTTTGCTACGA-3′; IL-1*β*, sense 5′-ACTGTGAAATGCCACCTTTTG-3′ and antisense 5′-TGTTGATGTGCTGCTGTGAG-3′; COX-2, sense 5′-AGGACTCTGCTCACGAAGGA-3′ and antisense 5′-TGACATGGATTGGAACAGCA-3′; MCP-1, sense 5′-GCTCTCTCTTCCTCCACCAC-3′ and antisense 5′-ACAGCTTCTTTGGGACACCT-3′; ICAM-1, sense 5′-CGCTTCCGCTACCATCAC-3′ and antisense GGCGGCTCAGTATCCTC-3′; VCAM-1, sense 5′-GGAGAGACAAAGCAGAAGTGG-3′ and antisense 5′-AACACAAGCGTGGATTTGG-3′.

### 2.6. Western Blotting

The total protein in the kidney tissue was extracted by a lysis buffer and the concentrations were determined through the Coomassie reagent. Equal amounts of the tissue protein (50 *μ*g) were denatured at 100°C for 10 min, separated by SDS-PAGE, and transferred onto nitrocellulose membranes. The blots were blocked overnight with 5% nonfat dry milk in Tris-buffered saline (TBS), followed by incubation for 1 h with rabbit polyclonal antibody, raised against mouse COX-2 and mPGES-1 (Cayman Chemical, MI, USA). The blots were washed with TBS followed by incubation with a horseradish peroxidase-conjugated secondary antibody and the immune complexes were detected using the ECL system (Amersham). The Bio-Rad electrophoresis image analyzer (Bio-Rad, UK) was used to quantify the protein signals.

### 2.7. Measurement of Renal PGE_2_ Content

The whole kidneys were snap-frozen in liquid nitrogen immediately upon collection and stored at −80°C. Then, the kidney tissues were lysed and homogenized, as previously described [13], and the PGE_2_ contents were measured using a commercially available PGE_2_ kit (Boshide, Biotech, China), according to the manufacturer's instructions.

### 2.8. Statistical Analysis

The values shown represent means ± SE. The data were analyzed by one-way analysis of variance (ANOVA), followed by a Bonferroni posttest. A *P* value < 0.05 was considered statistically significant.

## 3. Results

### 3.1. OA-NO_2_ Ameliorates Cisplatin-Induced Kidney Swelling

**Figure 1:**
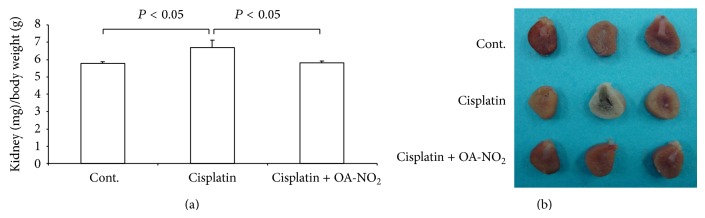
Pretreatment with OA-NO_2_ attenuated the kidney swelling (a) and improved the kidney appearance (b). Cont.: *N* = 6; cisplatin: *N* = 12; cisplatin + OA-NO_2_: *N* = 13. Data are mean ± SE.

Following the cisplatin treatment, the mice kidneys became pale and ischemic, in contrast to the control mice kidneys, whereas the pretreatment with OA-NO_2_ improved the kidney appearance (Figure 1(b)). The degree of kidney swelling secondary to the cisplatin-induced renal injury appeared more serious than that in the control mice group, while the pretreatment with OA-NO_2_ ameliorated the kidney swelling, as confirmed by the different kidney wet weight (cisplatin: 6.68 ± 0.42 versus control: 5.78 ± 0.11, *P* < 0.05; cisplatin: 6.68 ± 0.42 versus cisplatin + OA-NO_2_: 5.81 ± 0.08, *P* < 0.05) (Figure 1(a)).

### 3.2. OA-NO_2_ Ameliorates Cisplatin-Induced Renal Dysfunction

**Figure 2:**
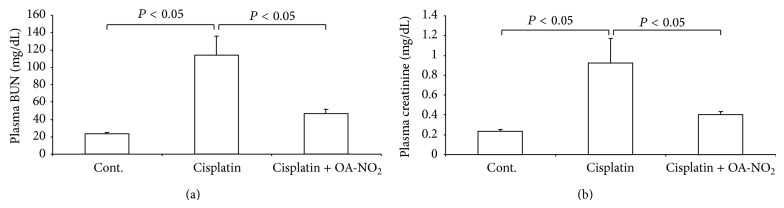
Plasma BUN (a) and creatinine (b) levels in control, cisplatin vehicle, and cisplatin + OA-NO_2_ mice. Cont.: *N* = 6; cisplatin: *N* = 12; cisplatin + OA-NO_2_: *N* = 13. Data are mean ± SE.

The renal function was assessed by the plasma BUN and creatinine levels. The cisplatin injection induced severe renal dysfunction, increasing plasma BUN from 23.3 ± 1.35 to 114.2 ± 22.1 mg/dL (*P* < 0.05) (Figure 2(a)) and plasma creatinine from 0.24 ± 0.02 to 0.93 ± 0.25 mg/dL (*P* < 0.05) (Figure 2(b)). Interestingly, the pretreatment with OA-NO_2_ resulted in reduced plasma BUN (46.7 ± 4.82 versus 114.2 ± 22.1 mg/dL, *P* < 0.05) (Figure 2(a)) and creatinine (0.40 ± 0.03 versus 0.93 ± 0.25 mg/dL, *P* < 0.05) levels, compared with the cisplatin group (Figure 2(b)).

### 3.3. OA-NO_2_ Attenuates Cisplatin-Induced Histological Changes in the Kidney

**Figure 3:**
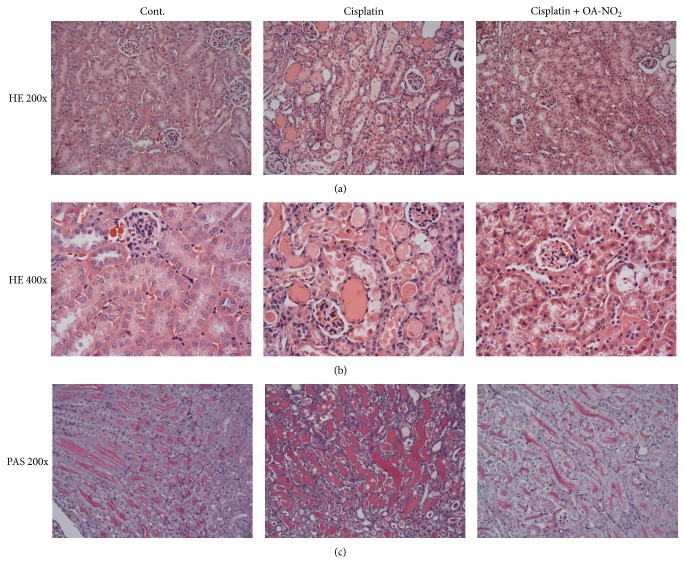
Morphological changes of cisplatin-induced renal injury in control, cisplatin vehicle, and cisplatin + OA-NO_2_ mice. ((a)–(c)) Representative photomicrographs with hematoxylin and eosin staining (magnification of 200 and 400) and periodic acid-Schiff staining (magnification 200) of renal cortex of kidneys; Cont.: *N* = 6; cisplatin: *N* = 12; cisplatin + OA-NO_2_: *N* = 13. Data are mean ± SE.

Following the cisplatin treatment, the mice in the cisplatin group displayed severe renal pathological changes, characterized by the distortion of the overall renal morphology, dilation of renal tubules, and appearance of protein cast, as well as severe tubular necrosis and interstitial inflammation. Moreover, the renal corpuscles displayed extensive congestion filling up the glomerular capillary loops, and some glomeruli were atrophied (Figures 3(a), 3(b), and 3(c)). Remarkably, these histological changes were alleviated after the pretreatment with OA-NO_2_. Indeed, the mice in the OA-NO_2_ pretreatment group exhibited considerably decreased semiquantitative histological damage scores, compared with those of the cisplatin group (Table 1).

### 3.4. Activation of Renal COX-2 by Cisplatin Treatment

**Figure 4:**
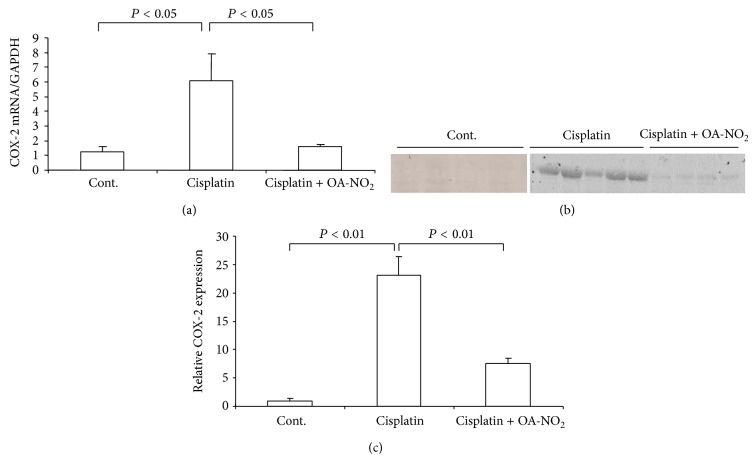
Renal COX-2 mRNA and protein expressions in control, cisplatin vehicle, and cisplatin + OA-NO_2_ mice. (a) qRT-PCR analysis of renal COX-2 mRNA; (b) western blotting analysis of renal COX-2 protein expression; (c) densitometric value of COX-2 protein; Cont.: *N* = 6; cisplatin: *N* = 12; cisplatin + OA-NO_2_: *N* = 13. Data are mean ± SE.

To determine the induction of COX-2 in the cisplatin-induced renal lesions, we examined COX-2 mRNA and protein levels in the kidneys after the cisplatin administration. COX-2 mRNA levels escalated strikingly (6.0-fold) after the cisplatin treatment. The pretreatment with OA-NO_2_ substantially reduced this increase (Figure 4). Similarly, western blotting showed that the COX-2 protein content was elevated 23.1-fold three days after the cisplatin treatment, and the pretreatment with OA-NO_2_ diminished this induction (Figure 4).

### 3.5. Activation of Renal mPGES-1 and PGE_2_ by Cisplatin Treatment

**Figure 5:**
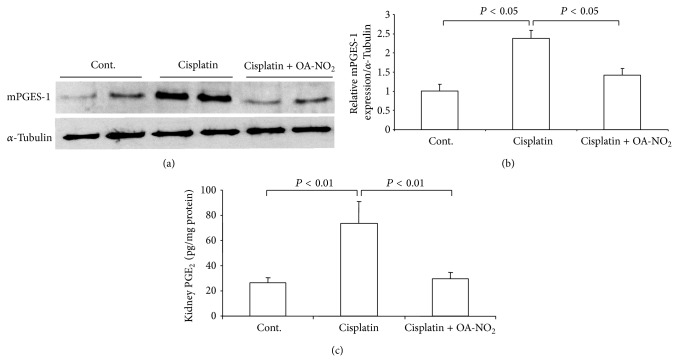
Renal mPGES-1 expression and renal PGE_2_ content in control, cisplatin vehicle, and cisplatin + OA-NO_2_ mice. (a) Immunoblot analysis of mPGES-1 expression; (b) densitometric value of mPGES-1 protein; (c) enzyme-linked immunosorbent assay analysis of renal PGE_2_ content. Cont.: *N* = 6; cisplatin: *N* = 12; cisplatin + OA-NO_2_: *N* = 13. Data are mean ± SE.

Next, we examined the mPGES-1 expression levels in the kidneys after the cisplatin administration. The mPGES-1 protein level markedly rose 2.1-fold after the cisplatin treatment. The pretreatment with OA-NO_2_ greatly lowered this increase (Figure 5(a)). To determine the COX-2/mPGES-1 cascade, we measured the kidney PGE_2_ content via enzyme-linked immunosorbent assay. At 72 h after the cisplatin treatment, the kidney PGE_2_ content was significantly enhanced in the cisplatin group mice. This augmentation was considerably blunted by the pretreatment with OA-NO_2_ (Figure 5(c)).

### 3.6. OA-NO_2_ Attenuates Cisplatin-Induced Renal Expression of Cytokines

**Figure 6:**
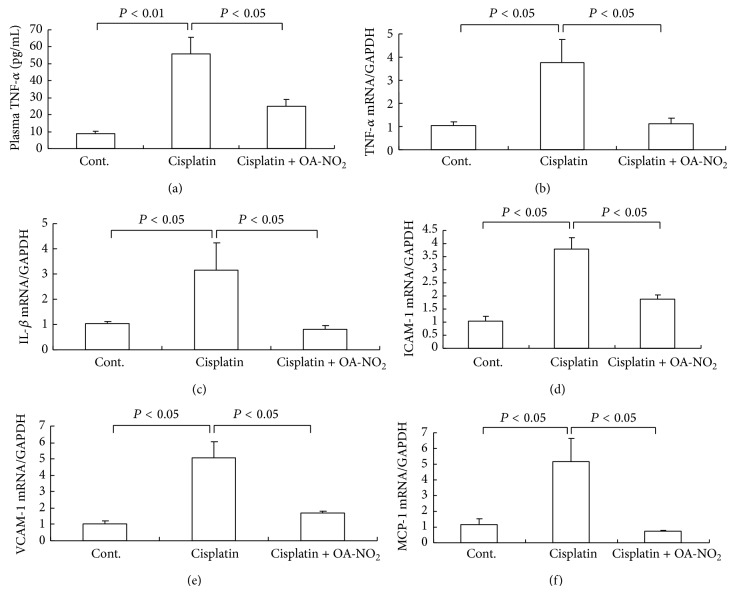
Renal proinflammatory cytokines and adhesion molecules in control, cisplatin vehicle, and cisplatin + OA-NO_2_ mice. (a) Enzyme-linked immunosorbent assay analysis of circulating TNF-*α*; the real-time RT-PCR of renal TNF-*α* mRNA (b), renal IL-1*β* mRNA (c), renal ICAM-1 mRNA (d), renal VCAM-1 mRNA (e), and renal MCP-1 mRNA (f) expression. Cont.: *N* = 6; cisplatin: *N* = 12; cisplatin + OA-NO_2_: *N* = 13. Data are mean ± SE.

TNF-*α* is a well-established pathogenic factor in the inflammatory responses in cisplatin-induced kidney injury. Therefore, the emphasis in our study was placed on the assessment of renal TNF-*α* mRNA and circulating TNF-*α* level. The renal TNF-*α* mRNA was increased 4.7-fold in the cisplatin-treatment group compared with the control mice group, and the pretreatment with OA-NO_2_ strikingly reduced the degree of TNF-*α* mRNA expressions (Figure 6(b)). Similarly, the circulating TNF-*α* level was elevated (6.2-fold) in the cisplatin-treated mice, while the elevation was attenuated by 50% after the pretreatment with OA-NO_2_ (Figure 6(a)). The renal interleukin- (IL-) 1*β* mRNA expression exhibited similar patterns as renal TNF-*α* (Figure 6(c)). Likewise, cisplatin induced the renal mRNA expression of chemokines and adhesion molecules, including the monocyte chemoattractant protein 1 (MCP-1), intercellular adhesion molecule-1 (ICAM-1), and vascular cell adhesion molecule-1 (VCAM-1); this effect was remarkably mitigated by the pretreatment with OA-NO_2_ (Figures 6(d)–6(f)). Overall, the pretreatment with OA-NO_2_ alleviated the expression of the proinflammatory cytokines, chemokines, and adhesion molecules in the cisplatin-induced kidney injury.

## 4. Discussion

Nitrooleic acid (OA-NO_2_) and its nitroalkene derivatives are robust endogenous ligands for the peroxisome proliferator-activated receptor-*γ* (PPAR*γ*), although their increased concentrations activate also PPAR*α* and PPAR*δ* [20, 21]. To date, OA-NO_2_ is established to have several properties, such as the therapeutic effects against dyslipidemia and hyperglycemia, associated with the metabolic syndrome. Moreover, the existing evidence indicates that OA-NO_2_ exerts potent anti-inflammatory actions.* In vitro*, it was determined that OA-NO_2_ inhibited the platelet aggregation, neutrophil activation, and nuclear factor *κ*B-mediated cytokine release and stimulated the heme oxygenase- (HO-) 1 expression in different inflammatory conditions [8, 22]. In animal models, OA-NO_2_ demonstrated a potential therapeutic effect in cardiac and renal ischemia and reperfusion via inhibiting the inflammatory mediator release and antioxidative stress [17, 23]. Nitrooleic acid attenuates colonic inflammation and improves the clinical symptoms in experimental inflammatory bowel disease [16]. In line with these observations, we previously revealed that the preventive treatment with OA-NO_2_ depressed the systemic and local inflammatory responses and improved the multiorgan dysfunction in septic mice. The effective anti-inflammatory effect of OA-NO_2_ appears to be attributable to the suppression of diverse proinflammatory mediators, including cytokines, chemokines, adhesion molecules, iNOS, and COX-2 [18]. In the present study, we established that the pretreatment with OA-NO_2_ attenuated the cisplatin-induced kidney injury in mice through the inhibition of COX-2/mPGES-1/PGE_2_ cascade expression and the suppressive action on other inflammatory cytokines.

The pathophysiological basis of cisplatin nephrotoxicity has been studied for the last three decades. However, only recently has the research been directed toward the understanding of its cellular and molecular mechanisms. Despite the intensive investigations, the processes underlying cisplatin nephrotoxicity are not fully understood. After administration, cisplatin is taken up in the renal tubular cells at high concentrations. The exposure of tubular cells to cisplatin activates complex signaling pathways that lead to the tubular cell injury and death. Meanwhile, robust inflammatory and oxidative stress responses are stimulated, further exacerbating the renal tissue damage. Cisplatin may also induce injury in the renal vasculature, leading to the ischemic tubular cell death and decreased glomerular filtration rate (GFR). These events accumulate and collectively culminate in the loss of renal functions during cisplatin nephrotoxicity, triggering acute renal failure. Among these multiple factors, inflammation plays the key role in the pathogenesis of cisplatin nephrotoxicity. It has been shown that cisplatin administration frequently results in the upregulation of proinflammatory mediators in the kidneys and activates the NF-*κ*B signaling pathway [24]. Among the inflammatory cytokines, the cyclooxygenase- (COX-) 2 has attracted more research attention and is considered to play an important role in mediating the cisplatin-induced renal injury [25].

Cyclooxygenase- (COX-) 2 is present throughout the different regions of the kidney and promotes the production of prostaglandins. It is involved in the constitutive production of prostanoids in the kidney and participates in the control of renal function and morphology. The most common action ascribed to COX-2 is the conversion of arachidonic acid to an intermediate endoperoxide that is converted to PGE_2_, PGI2, TXA2, or PGD_2_ through the actions of specific synthases [25]. Prostaglandin E2 (PGE_2_), a major product of arachidonic acid metabolism, has an established role in mediating pain and inflammatory responses [26]. To date, three major forms of PGES have been cloned and characterized: the membrane-associated PGES- (mPGES-) 1, mPGES-2, and cytosolic PGES [27]. Similarly to COX-2, mPGES-1 is highly induced and expressed in macrophages* in vitro* [28, 29] and in the spleen and the lungs* in vivo* [30, 31] in response to proinflammatory stimuli. In addition to the findings mentioned above, COX-2 also plays an important role in mediating the cisplatin-induced renal injury. In our previous study [32], a three-day cisplatin treatment (20 mg/kg) induced a marked widespread upregulation of mPGES-1 and COX-2 mRNA as well as of the protein levels and PGE_2_ content in the kidneys, while the renal expression of mPGES-2, cytosolic PGES, and COX-2 remained unaffected. However, the mPGES-1 KO mice were resistant to the cisplatin-induced renal dysfunction and structural damage, accompanied by suppressed inflammatory cytokine expression and oxidative stress. These results suggest that the activation of the COX-2/mPGES-1 pathway in the kidney may selectively mediate the cisplatin-induced nephrotoxicity. Consistently with these findings, another study [33] demonstrated the increase in COX-2 mRNA and strong labeling of COX-2 protein in the renal interstitial cells after a five-day cisplatin (13 mg/kg) treatment in mice, whereas the concomitant application of the selective inhibitor of COX-2 ameliorated the cisplatin-induced mouse renal lesions through the inhibition of inflammatory and oxidative stress responses. On the other hand, COX-1 mRNA levels were not affected by cisplatin treatment, suggesting no participation of COX-1-induced prostaglandins in cisplatin nephrotoxicity.

In the present study, the cisplatin administration induced both the COX-2 and mPGES-1 mRNA and protein levels, along with the increased renal PGE_2_ content. The pretreatment with OA-NO_2_ ameliorated the cisplatin-induced renal dysfunction and structural damage, accompanied by the suppressed expression of COX-2, mPGES-1, and other inflammatory cytokines. In spite of the already established anti-inflammatory effect of OA-NO_2_, we reported for the first time the capability of OA-NO_2_ to directly suppress the COX-2 expression in the context of inflammation [18]. The present study further determined that the nitrofatty acid inhibited the COX-2/mPGES-1 expression in the condition of inflammation. Overall, the intervention of COX-2/mPGES-1/PGE_2_ pathway by OA-NO_2_ in the cisplatin-treated mice may offer a novel approach for the management of the renal toxicity with added values of enhanced chemotherapeutic potential.

In addition to the COX-2/mPGES-1/PGE_2_ pathway, the renal and circulator levels of TNF-*α*, along with those of the renal interleukin-1*β* (IL-1*β*), renal monocyte chemoattractant protein 1 (MCP-1), renal intercellular adhesion molecule-1 (ICAM-1), and renal vascular cell adhesion molecule-1 (VCAM-1), are upregulated by cisplatin administration [7]. Besides the intervention in the COX-2/mPGES-1/PGE_2_ pathway, the pretreatment with OA-NO_2_ also suppressed diverse proinflammatory mediators, including TNF-*α*, IL-1*β*, MCP-1, ICAM-1, and VCAM-1. The beneficial effect of OA-NO_2_ in the cisplatin nephrotoxicity animal model is partially attributed to the suppression of these inflammatory mediators. The results obtained are consistent with those of the previous study, which demonstrated that renal TNF-*α* and IL-1*β* mRNA expression in the cisplatin-treated WT mice increased but were almost completely blocked in the mPGES-1 KO mice.

In summary, the present study evaluates* in vivo* the therapeutic effects of OA-NO_2_ in a mouse model of cisplatin-induced nephrotoxicity. The preventative treatment with OA-NO_2_ attenuated the cisplatin-induced renal dysfunction and structural damage. The beneficial effect of OA-NO_2_ appears to be attributable to the intervention in the COX-2/mPGES-1/PGE_2_ pathway and the suppression of proinflammatory mediators, including TNF-*α*, IL-1*β*, MCP-1, ICAM-1, and VCAM-1. Taken together, nitroalkenes may hold promise for prevention and possibly therapy of cisplatin-induced kidney injury.

## Figures and Tables

**Table 1:** Morphological changes of cisplatin-induced renal injury in control, cisplatin vehicle, and cisplatin + OA-NO_2_ mice as assessed by histological analysis.

Changes	Groups		
	Cont.	Cisplatin	Cisplatin + OA-NO_2_
Glomerular congestion	—	++	+
Glomerular atrophy	—	++	+
Cast	—	+++	+
Tubular brush border loss	—	++	+
Tubular necrosis	—	++	+
Tubular atrophy	—	+++	+
Interstitial inflammation	—	+++	+

Scoring scalescores (none (—); mild damage (+);  moderate damage (++); and severe damage (+++)) are semiquantitative scores given by a pathologist unaware of the type of treatment.
